# *Molecules* Best Paper Award 2014

**DOI:** 10.3390/molecules19011375

**Published:** 2014-01-22

**Authors:** Derek J. McPhee

**Affiliations:** Editor-in-Chief, MDPI AG, Klybeckstrasse 64, 2nd Floor, Basel CH-4057, Switzerland; E-Mail: mcphee@mdpi.com

*Molecules* instituted some years ago a “Best Paper” award to recognize the most outstanding papers in the area of natural products, medicinal chemistry and molecular diversity published each year in *Molecules*. We are pleased to announce the third “*Molecules* Best Paper Award” for 2014. The winners were chosen by the Editor-in-Chief and selected editorial board members from among all the papers published in 2010. Reviews and research papers were evaluated separately. We are pleased to announce that the following five papers have won the *Molecules* Best Paper Award in 2014:

**Article Award:**

*1^st^ Prize*

**Marco Felici, Pablo Contreras-Carballada, Jan M. M. Smits, Roeland J. M. Nolte, René M. Williams, Luisa De Cola and Martin C. Feiters**

Cationic Heteroleptic Cyclometalated Iridium^III^ Complexes Containing Phenyl-Triazole and Triazole-Pyridine Clicked Ligands

*Molecules* 2010, *15*(3), 2039–2059; doi:10.3390/molecules15032039

Available online: http://www.mdpi.com/1420-3049/15/3/2039

*2^nd^ Prize*

**Ahmad Asadinezhad, Igor Novák, Marián Lehocký, František Bílek, Alenka Vesel, Ita Junkar, Petr Sáha and Anton Popelka**

Polysaccharides Coatings on Medical-Grade PVC: A Probe into Surface Characteristics and the Extent of Bacterial Adhesion

*Molecules* 2010, *15*(2), 1007–1027; doi:10.3390/molecules15021007

Available online: http://www.mdpi.com/1420-3049/15/2/1007

*3^nd^ Prize*

**Marina Soković, Jasmina Glamočlija, Petar D. Marin, Dejan Brkić and Leo J. L. D. van Griensven**

Antibacterial Effects of the Essential Oils of Commonly Consumed Medicinal Herbs Using an *In Vitro* Model

*Molecules* 2010, *15*(11), 7532–7546; doi:10.3390/molecules15117532

Available online: http://www.mdpi.com/1420-3049/15/11/7532

**Review Award:**

*1^st^ Prize*

**Paul M. O’Neill, Victoria E. Barton and Stephen A. Ward**

The Molecular Mechanism of Action of Artemisinin—The Debate Continues

*Molecules* 2010, *15*(3), 1705-1721; doi:10.3390/molecules15031705

Available online: http://www.mdpi.com/1420-3049/15/3/1705

*2^nd^ Prize*

**Jin Dai and Russell J. Mumper**

Plant Phenolics: Extraction, Analysis and Their Antioxidant and Anticancer Properties

*Molecules* 2010, *15*(10), 7313-7352; doi:10.3390/molecules15107313

Available online: http://www.mdpi.com/1420-3049/15/10/7313

The prize awarding committee recognized the article “Cationic Heteroleptic Cyclometalated IridiumIII Complexes Containing Phenyl-Triazole and Triazole-Pyridine Clicked Ligands” as “…a nice experimental studies of luminescent probes…”. The article “Antibacterial Effects of the Essential Oils of Commonly Consumed Medicinal Herbs Using an In Vitro Model” was selected for its “…nice description of the chemical composition and antibacterial activity of essential oils from 10 commonly consumed herbs”. The review “The Molecular Mechanism of Action of Artemisinin—The Debate Continues” provided “…a very nice review on a timely topic, as the artemisinins are currently the major class of antimalarial drugs. It nicely summarizes the uncertainty surrounding the topic…” and “…utilizes the advantages of e-publication with photos and other illustrations. This is a well done review of worthwhile topics…”, and finally, the review “Plant Phenolics: Extraction, Analysis and Their Antioxidant and Anticancer Properties” provides “…an updated and comprehensive overview on phenolic extraction, purification, analysis and quantification as well as their antioxidant properties…”.

We believe these five exceptional papers represent valuable contributions to Molecules and the scientific literature. On behalf of the Prize Awarding Committee and the Editorial Board of Molecules, we would like to congratulate these five teams for their excellent work. In recognition for their accomplishment, Dr. Martin C. Feiters, Dr. Marián Lehocký and Dr. Leo J. L. D. van Griensven will receive prizes of 1,000, 800 and 600 CHF, respectively, and the privilege of publishing an additional open access format paper of their choice free of charge in Molecules. Dr. Paul M. O’Neill and Dr. Russell J. Mumper will be awarded the privilege of publishing an additional research paper free of charge in open access format in Molecules.

*Prize Awarding Committee*

*Editor-in-Chief*

**Dr. Derek J. McPhee**

MDPI AG, Klybeckstrasse 64, 2nd Floor, Basel CH-4057, Switzerland

E-Mail: mcphee@mdpi.com

*Editorial Board Member*, Section ‘Natural Products”

**Dr. John A. Beutler**

Molecular Targets Laboratory, Bldg 1052 Rm 110 NCI at Frederick, Frederick, MD 21702-1201, USA

E-Mail: beutlerj@mail.nih.gov

*Editorial Board Member*, Section ‘Medicinal Chemistry’

**Prof. Dr. Jarkko Rautio**

School of Pharmacy, University of Eastern Finland, P.O. Box 1627, 70211 Kuopio, Finland

E-Mail: jarkko.t.rautio@uef.fi

*Editorial Board Member*, Section ‘Organic Synthesis’

**Prof. Dr. Osmo E. O. Hormi**

Department of Chemistry, University of Oulu, Linnanmaa, P.O.Box 333, FIN-90570 Oulu, Finland

E-Mail: osmo.hormi@oulu.fi

